# ALG1-CDG Caused by Non-functional Alternative Splicing Involving a Novel Pathogenic Complex Allele

**DOI:** 10.3389/fgene.2021.744884

**Published:** 2021-09-09

**Authors:** Carlos Alberto González-Domínguez, Moisés O. Fiesco-Roa, Samuel Gómez-Carmona, Anke Paula Ingrid Kleinert-Altamirano, Miao He, Earnest James Paul Daniel, Kimiyo M. Raymond, Melania Abreu-González, Sandra Manrique-Hernández, Ana González-Jaimes, Roberta Salinas-Marín, Carolina Molina-Garay, Karol Carrillo-Sánchez, Luis Leonardo Flores-Lagunes, Marco Jiménez-Olivares, Anallely Muñoz-Rivas, Mario E. Cruz-Muñoz, Matilde Ruíz-García, Hudson H. Freeze, Héctor M. Mora-Montes, Carmen Alaez-Verson, Iván Martínez-Duncker

**Affiliations:** ^1^Laboratorio de Glicobiología Humana y Diagnóstico Molecular, Centro de Investigación en Dinámica Celular, Instituto de Investigación en Ciencias Básicas y Aplicadas, Universidad Autónoma del Estado de Morelos, Cuernavaca, Mexico; ^2^Instituto de Biotecnología, Universidad Nacional Autónoma de México, Cuernavaca, Mexico; ^3^Programa de Maestría y Doctorado en Ciencias Médicas, Universidad Nacional Autónoma de México (UNAM), Ciudad Universitaria, Mexico City, Mexico; ^4^Laboratorio de Citogenética, Instituto Nacional de Pediatría, Mexico City, Mexico; ^5^Centro de Rehabilitación e Inclusión Infantil Teletón, Tuxtla Gutiérrez, Mexico; ^6^Palmieri Metabolic Disease Laboratory, Children’s Hospital of Philadelphia, Philadelphia, PA, United States; ^7^Hospital Regional de Alta Especialidad Ciudad Salud, Tapachula, Mexico; ^8^Department of Laboratory Medicine and Pathology, Laboratory Genetics and Genomics, Mayo Clinic, Rochester, MN, United States; ^9^Genos Médica, Mexico City, Mexico; ^10^Laboratorio de Diagnóstico Genómico, Instituto Nacional de Medicina Genómica, Secretaría de Salud, Mexico City, Mexico; ^11^Laboratorio de Inmunología Molecular, Facultad de Medicina, Universidad Autónoma del Estado de Morelos, Cuernavaca, Mexico; ^12^Departamento de Neurología, Instituto Nacional de Pediatría, Mexico City, Mexico; ^13^Human Genetics Program, Sanford Burnham Prebys Medical Discovery Institute, La Jolla, CA, United States; ^14^Departamento de Biología, División de Ciencias Naturales y Exactas, Universidad de Guanajuato, Guanajuato, Mexico; ^15^Sociedad Latinoamericana de Glicobiología A.C, Cuernavaca, Mexico

**Keywords:** CDG, splicing, metabolic, glycosylation, ALG1, mutation, tetrasaccharide

## Abstract

This study reports on a Mexican mestizo patient with a multi-systemic syndrome including neurological involvement and a type I serum transferrin profile. Clinical exome sequencing revealed complex alleles in *ALG1*, the encoding gene for the chitobiosyldiphosphodolichol beta-mannosyltransferase that participates in the formation of the dolichol-pyrophosphate-GlcNAc2Man5, a lipid-linked glycan intermediate during *N*-glycan synthesis. The identified complex alleles were NM_019109.5(ALG1): c.[208 + 16_208 + 19dup; 208 + 25G > T] and NM_019109.5(ALG1): c.[208 + 16_208 + 19dup; 1312C > T]. Although both alleles carried the benign variant c.208 + 16_208 + 19dup, one allele carried a known *ALG1* pathogenic variant (c.1312C > T), while the other carried a new uncharacterized variant (c.208 + 25G > T) causing non-functional alternative splicing that, in conjunction with the benign variant, defines the pathogenic protein effect (p.N70S_S71ins9). The presence in the patient’s serum of the pathognomonic N-linked mannose-deprived tetrasaccharide marker for ALG1-CDG (Neu5Acα2,6Galβ1,4-GlcNAcβ1,4GlcNAc) further supported this diagnosis. This is the first report of an ALG1-CDG patient from Latin America.

## Introduction

Approximately 140 inborn errors of metabolism have been classified as congenital disorders of glycosylation (CDG), a rapidly expanding group of diseases caused by defects in the synthesis and attachment of glycans to glycoproteins and glycolipids (Ondruskova et al., 2021). ALG1-CDG is a subtype with severe multiorgan involvement (OMIM 608540) caused by pathogenic variants in *ALG1*. This gene encodes for a transmembrane chitobiosyldiphosphodolichol beta-mannosyltransferase that participates in the first steps of N-glycan biosynthesis that occur in the cytosolic side of the endoplasmic reticulum involving dolichol-pyrophosphate-GlcNAc2Man5 synthesis, an intermediate of the lipid-linked precursor oligosaccharide that is subsequently transferred to nascent glycoproteins for protein N-glycosylation. ALG1 extends GlcNAc2-PP-dolichol by adding the first mannose in ß1,4-linkage using GDP-mannose as a substrate donor to generate Man1GlcNAc2-PP-dolichol (Couto et al., 1984). ALG1 is part of a multi-mannosyltransferase complex that includes ALG1, ALG2, and ALG11 involved in a five-reaction process (Gao et al., 2004; O’Reilly et al., 2006; Aebi, 2013).

In contrast to PMM2-CDG, the most prevalent CDG affecting N-glycosylation with >1,000 patients reported worldwide, <80 cases of ALG1-CDG have been reported in the literature since 2004, none from Latin America (Grubenmann et al., 2004; Kranz et al., 2004; Schwarz et al., 2004; Dupré et al., 2010; Morava et al., 2012; Rohlfing et al., 2014; Barba et al., 2016; Harshman et al., 2016; Ng et al., 2016; Marques-da-Silva et al., 2017; Pérez-Cerdá et al., 2017; Medrano et al., 2019; Lei et al., 2020; Bogdañska et al., 2021; Quelhas et al., 2021). According to the Human Gene Mutation Database (HGMD), 50 disease-causing mutations have been reported in *ALG1*, including 41 missense/non sense, 6 splicing mutations, 1 small insertion, 1 small indel, and 1 gross deletion (as of July 2021).

Here, we describe a Mexican mestizo patient with ALG1-CDG who is a compound heterozygous of two pathogenic variants. One of these variants (NM_019109.5(ALG1):c.208 + 25G > T), previously uncharacterized, in combination with a known *cis* benign variant NM_019109.4(ALG1): c.208 + 16_208 + 19dup, causes, a new intronic donor splice site resulting in partial intron 1 retention (+27 bp), producing an aminoacid substitution and insertion (p.N70S_S71ins9).

## Patient Report

**FIGURE 1:**
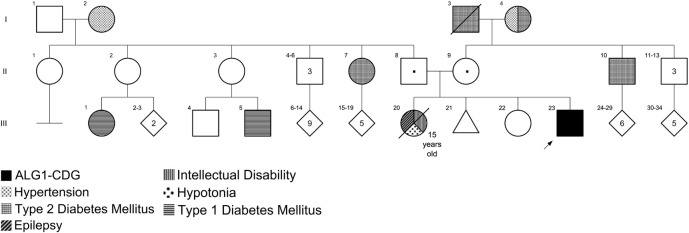
Pedigree of the family with ALG1-CDG. Individual III:23 is the patient reported in this work with ALG1-CDG. Individual III:20 had an intellectual disability, epilepsy, and hypotonia; however, genotyping was not possible because she died at 15 years old.

The patient was a 15-year-old male of Mexican ancestry with intellectual disability, seizures, hypotonic quadriplegia, and disproportionated microcephaly (height in normal centile). He was born in Tabasco State (in the southern part of Mexico). No consanguinity or endogamy was reported. The patient’s parents and an older sister were healthy, but he had another sister who died at 15 years old with similar manifestations. The mother reported a history of a first-trimester spontaneous abortion. There was no other relevant family history (Figure 1). The patient was the product of a naturally conceived 38-week singleton pregnancy to a 33-year-old mother and 37-year-old father, uneventful pregnancy with two normal prenatal ultrasounds. Delivery was via emergency cesarean section for prolonged rupture of membranes. At birth weight the patient was 3,100 g [46th centile; −0.1 standard deviation scores (SDS)] and length was 50 cm (50th centile; 0.5 SDS); additional birth parameters and APGAR score were unknown. He did not require respiratory support or supplementary interventions. He left the hospital 2 days after delivery. He was hospitalized for 30 days at 9 months of age due to seizures. During this hospitalization he was first noted to have some developmental delay; the mother reported the loss of some abilities after the onset of seizures.

He was first seen by a geneticist at 5 years old; however, no diagnosis was integrated. He was re-evaluated by geneticists at 10.8 years old. The anthropometric features were 32.3 kg of weight (24th centile; −0.71 SDS), 151 cm of height (85^th^ centile; + 1.05 SDS), and 49.2 cm of head circumference (< 3^rd^ centile; −3.01 SDS) (HP:0000252); the head circumference measurement at 4 years old was 46.3 cm (< 3^rd^ centile; −2.72 SDS), no previous head circumference evaluation was available. After a physical examination, the following findings were found: Short forehead (HP:0000350), eyebrow slightly sparse (HP:0000535), slightly prominent nasal bridge (HP:0000426), prominent cheeks (HP:0000293), wide mouth (HP:0000154), and widely spaced and misplaced teeth (HP:0000687 and HP:0006316, respectively). He had premature gray hair (HP:0002216) and one café-au-lait spot (HP:0000957) on his left forearm. The neurological evaluation found profound intellectual disability (HP:0002187), stereotypical and self-injurious behaviors (HP:0000733 and HP:0100716, respectively), sialorrhea (HP:0002307), central hypotonia (HP:0011398), hypotonic quadriplegia (HP:0002273), and generalized hyperreflexia (HP:0007034); no major motor milestones (gross motor function classification system level IV), and only guttural sounds [speech delay (alalia)] (HP:0001344). No other physical abnormality was detected.

**FIGURE 2:**
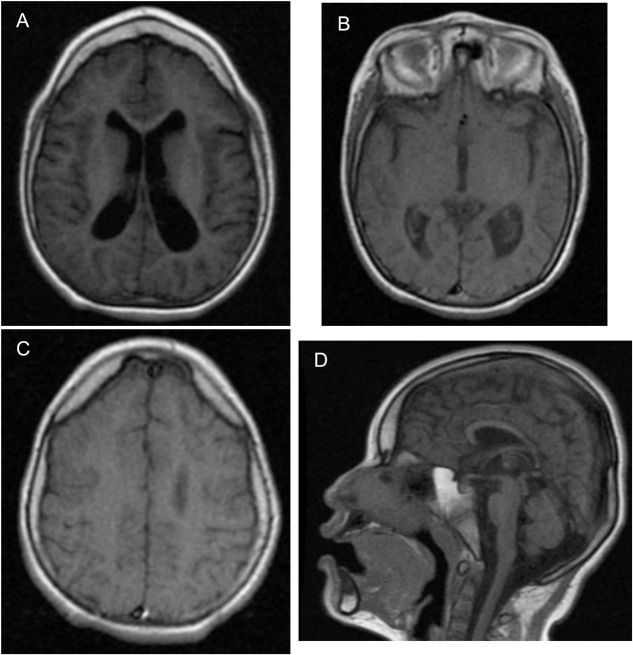
Abnormal brain MRI of the patient at 5 years old. **(A)** Cortical–subcortical frontotemporal atrophy, hypoplasia of the frontal lobes, and ventriculomegaly predominantly on the left on axial on axial T1-weighted FLAIR MR image. **(B)** Enlarged Sylvian cistern and ventriculomegaly predominantly on the left on axial T1-weighted MR image. **(C)** Frontotemporal cortical dysplasia pachygyria and polymicrogyria on axial T1-weighted MR image. **(D)** Hypoplastic *corpus callosum* and enlargement of *cisterna magna* without cerebellar atrophy on sagittal T1-weighted MR image.

During genetics follow-up, several investigations were performed. Metabolic screening for inborn errors of metabolism (including ammonia, lactate, plasma amino acids, and urine organic acids) was normal. Blood counts, INR, and prothrombin time were normal. No other laboratory tests were performed. An EEG performed at 10 years old showed diffuse slowing (HP:0010845), slow spike-and-wave activity (HP:0010850), and frontal intermittent rhythmic delta activity (FIRDA). The visual and brainstem-auditory evoked potentials revealed bilateral integrity of visual and auditory pathways, respectively. MRI was abnormal (Figure 2), showing generalized and asymmetric cortical-subcortical frontotemporal atrophy (HP:0006892) predominantly on the left, hypoplasia of the frontal lobes (HP:0007333), enlarged Sylvian cistern (HP:0100952), frontotemporal cortical dysplasia [pachygyria (HP:0001302) and polymicrogyria (HP:0002126)], asymmetric temporo-occipital ventriculomegaly (HP:0006956) predominantly on the left, hypoplastic *corpus callosum* (HP:0002079), and enlargement of *cisterna magna* (HP:0002280) without cerebellar atrophy. For the phenotype description, the Human Phenotype Ontology was used (Köhler et al., 2021).

## Results

**FIGURE 3:**
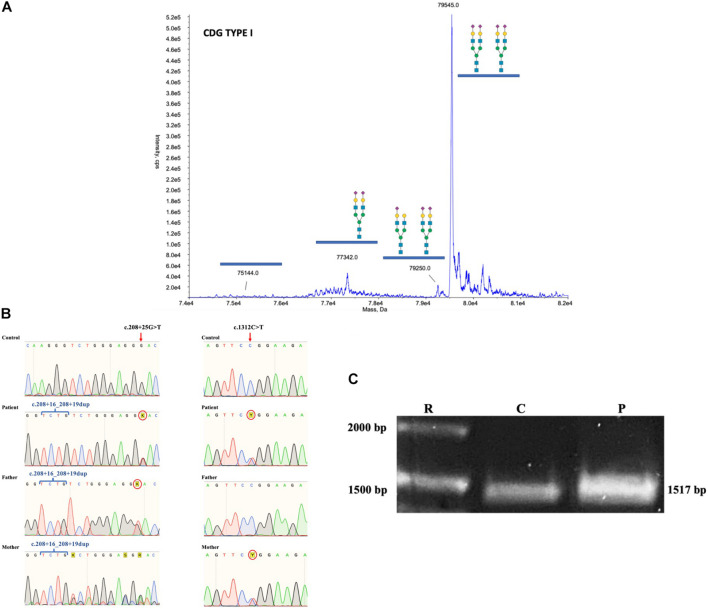
**(A)** ESI-MS analysis of serum Tf isoforms. Profile of the patient showing an increase of mono-oligo Tf, peaks are annotated with the glycosylation moiety structure with the following meaning: blue box = N-acetylglucosamine; green circle = mannose; yellow circle = galactose; purple diamond = N-acetylneuraminic acid (sialic acid). **(B)** Chromatograms of gDNA sequencing from healthy control, mother, father, and patient showing the inheritance of the *ALG1* c.208 + 16_208 + 19dup, the c.208 + 25G > T, and the c.1312C > T variants. **(C)** PCR of the coding sequence of *ALG1* showing the expected wt 1517 bp size in healthy control and the patient’s amplicon showing a slightly thicker band.

Based on the clinical phenotype, electrospray ionization mass spectrometry (ESI-MS) analysis of serum transferrin (Tf) was performed and a type I profile was established (mono-oligo/di-oligo = 0.90) (Figure 3A). To determine the genetic basis of the disease, a skin biopsy was performed to obtain a fibroblast culture. The genomic DNA (gDNA) obtained from the patient’s fibroblasts was sent to clinical exome sequencing (CES), and three *ALG1* variants were found in the form of two complex alleles: one complex allele composed by a benign variant NM_019109.4(ALG1): c.208 + 16_208 + 19dup in *cis* with the known pathogenic variant NM_019109.5(ALG1):c.1312C > T (p.R438W) defined as NM_019109.5(ALG1): c.[208 + 16_208 + 19dup; 1312C > T] and a second complex allele composed by the same benign variant NM_019109.4(ALG1): c.208 + 16_208 + 19dup in *cis* with a previously not described variant c.208 + 25G > T that we termed NM_019109.5(ALG1): c.[208 + 16_208 + 19dup; 208 + 25G > T].

Sequencing of the parents’ gDNA showed that the c.[208 + 16_208 + 19dup; 1312C > T] complex allele was inherited from the mother and the c.[208 + 16_208 + 19dup; 208 + 25G > T] from the father (Figure 3B). The Sanger sequencing of the mother’s *ALG1* shows an overlap that is the result of heterozygosity for the benign c.208 + 16_208 + 19dup variant, in contrast to the father that is homozygote.

**FIGURE 4:**
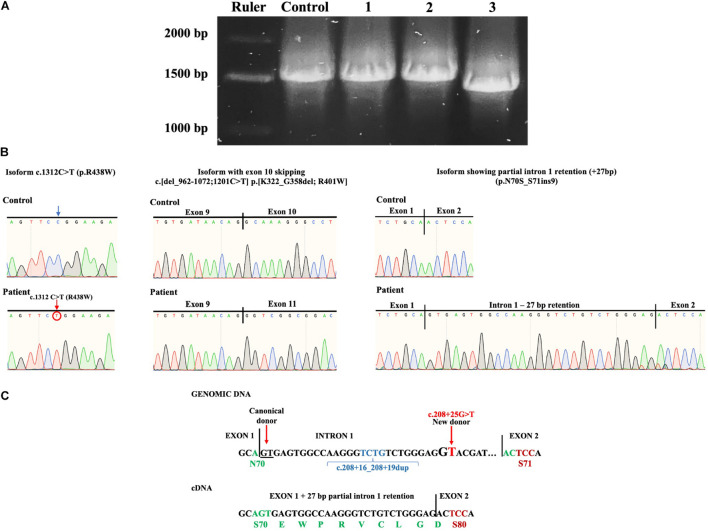
**(A)** Isoforms found after PCR subcloning of the patient’s *ALG1* amplicon. R, ruler; C, healthy wt control; 1, a constitutively spliced isoform bearing the c.1312C > T (p.R438W) variant; 2, an alternatively spliced isoform with partial intron 1 retention (+27bp); 3, an alternatively spliced isoform bearing the c.1312C > T variant with exon 10 skipping c.[del_962-1072;1201C > T] p.[K322_G358del; R401W]. **(B)** Sanger sequencing chromatograms of patient’s isoforms showing the c.1312C > T (p.R438W) variant; exon 10 skipping c.[del_962-1072;1201C > T] p.[K322_G358del; R401W] and partial intron 1 retention (+ 27 bp) (p.N70S_S71ins9). **(C)** Splicing mechanism that induces the + 27 bp partial intron 1 retention caused by the complex allele c.[208 + 16_208 + 19dup; 208 + 25G > T]. The new donor splicing site (GG to GT) causes partial intron 1 retention (+ 27 bp) that when translated would result in aminoacid substitution N70S and insertion of nine aminoacids.

A consideration in the case of the complex allele c.[208 + 16_208 + 19dup; 208 + 25G > T] is that the c.208 + 25G > T is located + 29 bp from the donor splicing site in exon 1 due to a four base duplication (TCTG) caused by the benign variant c.208 + 16_208 + 19dup (chr16:5122072); ClinVar Variation ID 95935, dbSNP:rs35400794 (Figure 4C).

The c.208 + 25G > T variant was considered potentially pathogenic because it could induce a new donor splice site (GG to GT) and cause non-functional alternative splicing. Using the Human Splicing Finder prediction (HSFPro, Genomnis), it was found that this change significantly alters splicing with the following values [WT-Mut%variation] [HSF Donor site (matrix GT) 61.35 > 88.49 (44.24%) and MaxEnt Donor site 1.01 > −8.65 (756.44%)].

To further investigate the presence of alternative splicing, the patient’s complementary DNA (cDNA) was synthesized and *ALG1* was polymerase chain reaction (PCR) amplified. The *ALG1* has 13 exons and codes for a 464 aminoacid protein. Amplification of *ALG1* with primers ALG1s and ALG1as encompasses the coding sequence plus short stretches from the 5′- and 3′-UTR for a predicted amplicon of 1517 bp. PCR product analysis in an agarose gel showed a slightly thicker amplicon in the patient compared to the healthy control (Figure 3C). Because this suggested potential splicing isoforms in the patient, subcloning and screening were performed. Three types of isoforms were identified: one with the c.1312C > T variant and constitutive splicing (c.1312C > T; p.R438W) or with the same variant (shifted to position 1201) and exon 10 skipping c.[del_962-1072;1201C > T] translated as p.[K322_G358del; R401W] and a third isoform without the c.1312C > T variant, but that presented partial intron 1 retention (+ 27 bp) without causing a frameshift and that theoretically results when translated in substitution of N70S and insertion of nine aminoacids EWPRVCLGD (p.N70S_S71ins9) (Figures 4A–C).

**FIGURE 5:**
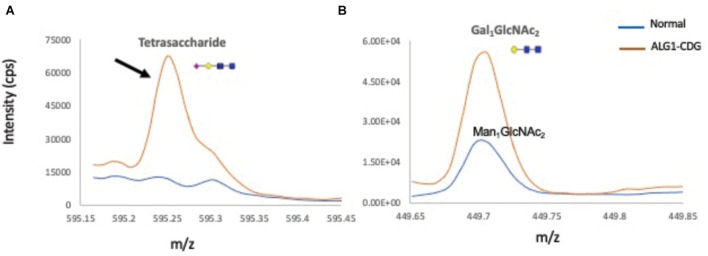
Total ion chromatograms of N-tetrasaccharide (NeuAc1Gal1GlcNAc2) and Gal1 or Man1GlcNAc2 in serum N-glycan profiles from a normal control and the ALG1-CDG patient. Overlay of total ion chromatograms of N-linked mannose deprived tetrasaccharide **(A)**, Man1/Gal1GlcNAc2 **(B)** of a normal control plasma (in blue), and ALG1-CDG (red). Gal1GlcNAc2 is absent in total plasma proteins from normal controls, which instead have traces of Man1GlcNAc2 present. Markedly increased tetrasaccharide was detected in the ALG1-CDG patient as shown with a black arrow.

To further biochemically confirm the ALG1-CDG diagnosis, previous works have shown that ALG1-CDG patients accumulate a novel N-linked mannose-deprived tetrasaccharide considered as a pathognomonic marker for ALG1-CDG (Neu5Acα2,6Galβ1,4-GlcNAcβ1,4GlcNAc) on serum glycoproteins (Bengtson et al., 2016; Ng et al., 2016; Zhang et al., 2016; Chen et al., 2019). Total serum N-gycans were analyzed by electrospray ionization–quadrupole time-of-flight mass spectrometry (ESI-QTOF) to detect the presence of the mannose-deprived N-tetrasaccharide, finding it significantly elevated at 0.23% of total glycan (normal 0–0.03); Gal1GlcNAc2 was also increased at 0.12% (normal 0) (Figure 5).

## Discussion

This report presents a patient with a severe clinical multisystemic phenotype with neurological involvement and an ESI-MS Tf with a type I profile consistent with CDG. CES revealed two complex alleles: The NM_019109.5(ALG1): c.[208 + 16_208 + 19dup; 1312C > T], inherited from the mother, and the NM_019109.5(ALG1): c.[208 + 16_208 + 19dup; 208 + 25G > T] inherited from the father. We considered that the latter could cause non-functional alternative splicing as predicted by HSFPro (Genomnis). PCR-based analysis of the patient’s *ALG1* amplicon further supported this hypothesis (Figure 3C).

To determine the effects of the complex alleles, the patient’s *ALG1* PCR amplicon was subcloned and screened, identifying three types of isoforms (Figure 4A), two derived from the c.[208 + 16_208 + 19dup; 1312C > T] complex allele and the third isoform with partial intron 1 retention (+ 27 bp) that did not present the c.1312C > T variant and that we conclude is derived from the c.[208 + 16_208 + 19dup; 208 + 25G > T] complex allele.

Regarding the isoforms derived from the c.[208 + 16_208 + 19dup; 1312C > T] complex allele, inherited by the mother, one presented constitutive splicing (p.R438W) and another an unexpected alternative splicing (exon 10 skipping) that translates into a 427 aa protein lacking 37 aminoacids p.[K322_G358del;R401W]. Because no variants were found in intron/exon junctions of exon 10, we consider that the c.1312C > T variant located in exon 13 could be responsible for altering distant splicing events, although further analysis is required to make a definitive conclusion. The c.1312C > T pathogenic variant included in this complex allele has been reported in six patients as compound heterozygotes with the following pathogenic variants, c.866A > G, c.450C > A, c.1145T > C, and c.1236A > G, and has been demonstrated to have reduced function (Dupré et al., 2010; Rohlfing et al., 2014; Ng et al., 2016; Zhang et al., 2016). No deleterious effects due to the benign intronic variant were observed.

For the third isoform without the c.1312C > T variant and with partial intron 1 retention (+ 27 bp) we conclude that it results from the paternally inherited complex allele c.[208 + 16_208 + 19dup; 208 + 25G > T] where the c.208 + 25G > T variant induces a new donor splice site that causes partial intron 1 retention (+ 27 bp). The partial intron 1 retention does not cause a frameshift but results in aminoacid substitution (N70S) and insertion of nine aminoacids (EWPRVCLGD) (p.N70S_S71ins9) (Figure 4C). It is important to note that the duplication of the TCTG bases occurring at the c.208 + 16_208 + 19dup variant (chr16:5122072) shifts the position c.208 + 25G > T to + 29 bp from the donor splicing site in exon 1 which determines the length of intron retention and preservation of the ORF (Figure 4C). We consider that the resulting p.N70S_S71ins9 has a reduced function based on the different physicochemical properties at position 70 [acidic (N) to hydroxylic (S)] as well as the insertion of nine aminoacids, including a P residue. Also, a previously reported p.S71F variant has been shown to have reduced function (Ng et al., 2016).

The presence of the ALG1-CDG pathognomonic N-linked mannose-deprived tetrasaccharide detected by ESI-QTOF when analyzing serum total N-glycans further supports the conclusion that the resulting protein variant p.N70S_S71ins9 does not have a normal function as it is not able to compensate for the decreased function of the p.R438W pathogenic variant. The fact that the N-tetrasaccharide was not detected by ESI-MS of serum Tf (Figure 3A) could be explained by differential sensitivity related to types of equipment and techniques, as well as a possible increased sensitivity to detect the N-tetrasaccharide when analyzing total N-glycans versus Tf glycans alone.

It is noteworthy that the c.208 + 16_208 + 19dup variant is considered a benign variant but that the additional presence of the c.208 + 25G > T variant results in a pathogenic complex allele c.[208 + 16_208 + 19dup; 208 + 25G > T]. According to gnomAD database (Lek et al., 2016) the variant c.208 + 16_208 + 19dup is very frequent in all ethnic groups (gnomAD ExomesVersion: 2.1.1 global frequency *f* = 0.558), as of July 2021. Concerning the c.208 + 25G > T variant, it is absent from the genomes and exomes in the genomAD database (as of July 2021). Interestingly, in the absence of the c208 + 16_208 + 19dup, the c.208 + 25G > T variant would cause a frameshift and a premature stop codon in exon 2 which would also be pathogenic and probably more severe.

Given these results, ALG1-CDG diagnosis was clinically, biochemically, and genetically established. In most disease-related genes, variants affecting splicing are not fully characterized because variant screening is restricted to gDNA. In our experience involving ATP6V0A2-CDG and more recently PMM2-CDG, amplification of cDNA transcripts is an invaluable tool to demonstrate non-functional alternative splicing, identifying the consequence on the protein and establishing the pathogenicity mechanism (Bahena-Bahena et al., 2014; González-Domínguez et al., 2020, 2021). Of the six reported disease-causing mutations in HGMD that potentially affect splicing of *ALG1*, only one has been experimentally confirmed (Table 1). The c.208 + 25G > T reported in this work would be the second variant confirmed to cause alternative non-functional splicing.

**TABLE 1:** Variants of *ALG1* affecting splicing classified as disease-causing mutations in the Human Gene Mutation Database (HGMD) and the novel variant reported in this work.

Variants	Splicing site affected	Data confirming splicing	References
c.208 + 25G > T	Donor intron 1	Partial intron 1 retention	This work
c.209-1G > C	Acceptor intron 1	No	Ng et al., 2016
c.961 + 1G > C	Donor intron 9	No	Ng et al., 2016
c.1187 + 1G > A	Donor intron 11	No	Jones et al., 2013; Ng et al., 2016
c.1187 + 3A > G	Donor intron 11	Intron 11 retention	Ng et al., 2016; Bowling et al., 2017
c.1188-2A > G	Acceptor intron 11	No	Ng et al., 2016
c.1188T > A (p.Cys396Ter)	Acceptor intron 11	No	Dupré et al., 2010

This case also highlights the importance of increasing awareness and availability of technological platforms for biochemical and genetic diagnosis of rare diseases. This patient and his family had to wait 15 years to obtain a diagnosis, with a sibling who died at the same age without diagnosis. This time to diagnosis is not adequate for ALG1 patients that have an estimated premature death rate of 44% of which 65% occur at < 12 months of age (Ng et al., 2016). This is a lengthy diagnostic odyssey that is unfortunately too frequent in Latin American and most underdeveloped countries that in the era of exome sequencing can no longer be acceptable. It is necessary that academic and family organizations around the world pressure for a shift in global health public policy to guarantee that all patients affected by rare diseases have access to an interdisciplinary approach as well as free exome sequencing (Manickam et al., 2021).

## Conclusion

The complex allele c.[208 + 16_208 + 19dup; 208 + 25G > T] (p.N70S_S71ins9) is pathogenic by causing non-functional alternative splicing of ALG1. Variants should be studied concerning their potential disruption of splicing, particularly if they affect canonical splicing sites. Increased awareness of rare diseases, including CDGs as well as the availability of technological platforms for genetic diagnosis, must be an international standard in public health policy.

## Materials and Methods

### Informed Consent

Informed consent was obtained from both parents to perform a skin biopsy, fibroblast cultures, and all required research to obtain a molecular diagnosis and to publish other data on the patient and parents. All procedures followed were in accordance with national and institutional ethical standards on human experimentation and with the Helsinki Declaration of 1975 and revised in 2000.

### Electrospray Ionization Mass Spectrometry (ESI-MS) Analysis of Serum Transferrin (Tf)

On-column immunoaffinity ESI-MS analysis of serum Tf isoforms was performed using an API-5000 triple quadrupole mass spectrometer (Applied Biosystems/MDS Sciex, Foster City, CA, United States).

### Analysis of N-Linked Mannose Deprived-Tetrasaccharide

The patient’s serum total N-glycans were analyzed by ESI-QTOF to detect the (Neu5Acα2,6Galβ1,4-GlcNAcβ1,4GlcNAc) N-tetrasaccharide as previously described (Chen et al., 2019).

### Cell Culture

From the patient’s skin biopsy, a primary culture of fibroblasts was obtained in AmnioMAX^TM^ C-100 Basal Medium (Gibco^®^ by Life Technologies^TM^; Life Technologies, Rockville, MD, United States) supplemented with 15% AmnioMAX^TM^ C-100 Supplement (Gibco^®^ by Life Technologies^TM^) and 1% penicillin/streptomycin antibiotic (Gibco^®^ by Life Technologies^TM^). Fibroblast cultures were maintained at 37°C in a humidified atmosphere containing 5% CO_2_. Fibroblasts were further processed to obtain genetic material.

### Clinical Exome Sequencing (CES)

The gDNA was extracted from fibroblasts using Maxwell^®^ 16 Blood DNA Purification Kit (Promega, Madison, WI, United States). The purity and concentration of the DNA samples were measured using NanoDrop 1000 spectrophotometer (Thermo Fisher Scientific, Waltham, MA, United States) and Qubit fluorometer (Thermo Fisher Scientific). Library preparation was performed using the reagents provided in the Clinical Exome sequencing panel kit, version 2 (Sophia Genetics SA, Saint Sulpice, Switzerland), according to the manufacturer’s protocol. Sequencing was performed on NextSeq Instrument (Illumina, San Diego, CA, United States). Sequencing data analysis and variant annotation were performed with the Sophia DDM^®^ software version 5.9.1.1 (Sophia Genetics SA). A bioinformatic filter was constructed, including all the genes previously reported to be related to CDG.

### Predictions of the Pathogenicity of the Variants

The HSFpro software (Genomnis; Desmet et al., 2009) was used to predict the effect of variants on splicing. All predictions were made with the DYSF transcript ENST00000268261.

### Polymerase Chain Reaction (PCR) and Sanger Sequencing

Total mRNA from the patient’s fibroblasts was obtained using TRIzol reagent (Life Technologies) and cDNA was synthesized using M-MLV Reverse Transcriptase (Life Technologies). The cDNA-based PCR product corresponding to the coding sequence of *ALG1* was obtained using forward primer ALG1s 5′-TGACTGCTGCGGGCCAG-3′ and reverse primer ALG1as 5′-CACTGGGAGGTGCTGCTCG-3′. In the case of the patient, the amplicon was isolated in low melting point agarose gel, purified, subcloned, and screened for alternative splicing.

The inheritance of variants was determined by analyzing the patient’s and parents’ gDNA using primer ALG1s and reverse primer ALG1gas 5′-CTAAAGGAGCACTTCCGCC-3′ for the c.208 + 25G > T pathogenic variant and forward primer ALG1-E13s 5′-CAGGCAATGAGGTAAGCTCTG-3′ and reverse primer ALG1-E13as 5′-CAATTCTTTTACCAGGCAGTACC-3′ for the c.1312C > T pathogenic variant. Sequencing was performed by an ABI Prism 3130xl autoanalyzer (Applied Biosystems, Foster City, CA, United States), and results were visualized using SnapGene Viewer 2.2.2 (GSL Biotech LLC, Chicago, IL, United States).

## Data Availability Statement

The novel pathogenic variant observed in this study has been deposited in the ClinVar database with the accession SCV001761655.

## Ethics Statement

The studies involving human participants were reviewed and approved by the Ethics Committee of the Sociedad Latinoamericana de Glicobiología. Written informed consent to participate in this study was provided by the participants’ parents.

## Author Contributions

IM-D and CG-D conceived and designed the study. CG-D, MF-R, SG-C, AK-A, MH, ED, KR, MA-G, SM-H, AG-J, RS-M, CM-G, KC-S, LF-L, MJ-O, AM-R, MC-M, MR-G, HH-F, and CA-V contributed to data acquisition and analysis. IM-D, CG-D, CA-V, HH-F, and HM-M contributed to curation and interpretation of data. HM-M and IM-D: provided supervision. IM-D wrote the original draft. CG-D, MF-R, SG-C, A-KA, MH, ED, KR, M-AG, SM-H, AG-J, RS-M, CM-G, KC-S, LF-L, MJ-O, AM-R, MC-M, MR-G, HM-M, HH-F, CA-V, and IM-D revised, edited, and approved the final version of the manuscript.

## Conflict of Interest

The authors declare that the research was conducted in the absence of any commercial or financial relationships that could be construed as a potential conflict of interest.

## Publisher’s Note

All claims expressed in this article are solely those of the authors and do not necessarily represent those of their affiliated organizations, or those of the publisher, the editors and the reviewers. Any product that may be evaluated in this article, or claim that may be made by its manufacturer, is not guaranteed or endorsed by the publisher.
