# Corrigendum: ALG1-CDG Caused by Non-Functional Alternative Splicing Involving a Novel Pathogenic Complex Allele

**DOI:** 10.3389/fgene.2021.777731

**Published:** 2021-09-30

**Authors:** Carlos Alberto González-Domínguez, Moisés O. Fiesco-Roa, Samuel Gómez-Carmona, Anke Paula Ingrid Kleinert-Altamirano, Miao He, Earnest James Paul Daniel, Kimiyo M. Raymond, Melania Abreu-González, Sandra Manrique-Hernández, Ana González-Jaimes, Roberta Salinas-Marín, Carolina Molina-Garay, Karol Carrillo-Sánchez, Luis Leonardo Flores-Lagunes, Marco Jiménez-Olivares, Anallely Muñoz-Rivas, Mario E. Cruz-Muñoz, Matilde Ruíz-García, Hudson H. Freeze, Héctor M. Mora-Montes, Carmen Alaez-Verson, Iván Martínez-Duncker

**Affiliations:** ^1^ Laboratorio de Glicobiología Humana y Diagnóstico Molecular, Centro de Investigación en Dinámica Celular, Instituto de Investigación en Ciencias Básicas y Aplicadas, Universidad Autónoma del Estado de Morelos, Cuernavaca, Mexico; ^2^ Instituto de Biotecnología, Universidad Nacional Autónoma de México, Cuernavaca, Mexico; ^3^ Programa de Maestría y Doctorado en Ciencias Médicas, Universidad Nacional Autónoma de México (UNAM), Ciudad Universitaria, Mexico City, Mexico; ^4^ Laboratorio de Citogenética, Instituto Nacional de Pediatría, Mexico City, Mexico; ^5^ Centro de Rehabilitación e Inclusión Infantil Teletón, Tuxtla Gutiérrez, Mexico; ^6^ Hospital Regional de Alta Especialidad Ciudad Salud, Tapachula, Mexico; ^7^ Palmieri Metabolic Disease Laboratory, Children’s Hospital of Philadelphia, Philadelphia, PA, United States; ^8^ Department of Laboratory Medicine and Pathology, Laboratory Genetics and Genomics, Mayo Clinic, Rochester, MN, United States; ^9^ Genos Médica, Mexico City, Mexico; ^10^ Laboratorio de Diagnóstico Genómico, Instituto Nacional de Medicina Genómica, Secretaría de Salud, Mexico City, Mexico; ^11^ Laboratorio de Inmunología Molecular, Facultad de Medicina, Universidad Autónoma del Estado de Morelos, Cuernavaca, Mexico; ^12^ Departamento de Neurología, Instituto Nacional de Pediatría, Mexico City, Mexico; ^13^ Human Genetics Program, Sanford Burnham Prebys Medical Discovery Institute, La Jolla, CA, United States; ^14^ Departamento de Biología, División de Ciencias Naturales y Exactas, Universidad de Guanajuato, Guanajuato, Mexico; ^15^ Sociedad Latinoamericana de Glicobiología A.C, Cuernavaca, Mexico

**Keywords:** CDG, splicing, metabolic, glycosylation, ALG1, mutation, tetrasaccharide

In the published article, there were errors regarding the affiliations for Anke Paula Ingrid Kleinert-Altamirano, Earnest James Paul Daniel, and Miao He.

Anke Paula Ingrid Kleinert-Altamirano should have affiliations 5 “Centro de Rehabilitación e Inclusión Infantil Teletón, Tuxtla Gutiérrez, Mexico” and 7 “Hospital Regional de Alta Especialidad Ciudad Salud, Tapachula, Mexico” instead of 5 and 6.

Miao He and Earnest James Paul Daniel should have affiliation 6 “Palmieri Metabolic Disease Laboratory, Children’s Hospital of Philadelphia, Philadelphia, PA, United States” instead of 7.

The authors apologize for these errors and state that this does not change the scientific conclusions of the article in any way. The original article has been updated.

